# Medical Opinion and Movement

**Published:** 1906-10-27

**Authors:** 


### Medical Opinion and Movement.

As we have already pointed out, the remedy for existing hospital abuse, due to granting free relief to practically all who apply for it at the hospitals, is a combined action on the part of the general practitioners with the object of bringing pressure to bear upon the members of the visiting staffs of the voluntary hospitals, through whom alone an efficient remedy can be effectually applied. It is interesting in this connection to notice that the lay governors of hospitals show an increasing tendency to put the responsibility of admitting a patient to treatment at a voluntary hospital more and more into the hands of the visiting medical staff. This tendency was shown in a practical way by the governors of the Norfolk and Norwich Hospital. At the last quarterly court, after a full discussion, it was determined by a majority of nearly four to one, despite strong opposition on the part of representatives of the Hospital Sunday and Saturday Funds and others, that out-patient tickets should be abolished altogether. We shall watch with interest the effects of this decision upon the finances of the Norfolk and Norwich Hospital. Meanwhile it enforces our contention that the speediest and, in effect, the only practical remedy for the present excessive free medical relief is to compel united action on the part of the visiting medical staff to secure its removal.

As showing the trend of medical opinion amongst some members of the profession, it may be interesting to record that a meeting of medical men of the Witwatersrand, Tranvsaal, has decided with practical unanimity to establish a medical union which will deal, amongst other things, with the subject of contract medical practice. A resolution was proposed which declared, in effect, that except for paupers and whole-time appointments contract practice is not necessary. After a prolonged debate this resolution was withdrawn in favour of one establishing a medical union. We fully recognise the difficulties of general practitioners in view of modern developments and the general extension of hospitals. It is, however, most desirable that a learned profession like that of medicine should avoid committing itself to anything which partakes of the nature of a trades union. If the public once get it into their heads that the ordinary practitioner places the payment of his fee before every other consideration, the present influence of the medical profession will be undermined, and its usefulness greatly impaired. That is why the recent proceedings of the British Medical Association in various directions warrant the most earnest consideration of every practitioner who values the honour of his profession and is anxious to maintain alike its best traditions, his own authority, and the general good of the whole community.

Mr. Sidney Webb has done good service by publishing two papers in the *Times* on the diminishing birth-rate. The statistics which he has been able to produce are incomplete and comparatively few, but their drift is reasonably conclusive, and they fairly indicate some of the principal causes to which the danger is due. Mr. Sidney Webb properly insists that some care and adequate precaution should be taken to protect the newly-born infant. He urges the institution of a systematic endowment of motherhood. This would include free medical attendance both for the child-bearing mother and the children; the supply of milk by the municipality to all infants, and the right of the mother, whilst nursing her baby, to free meals. Mr. Sidney Webb further urges the provision of free meals for school children, and the institution of a large number of maintenance scholarships for secondary, technical, and university education and as an encouragement to larger families amongst members of the middle and upper artizan classes. He is further strongly in favour of the State providing higher schools and colleges, either free or at nominal rates, for the education of the children of these classes. The whole question is one which demands the most careful attention of Statesmen and members of the profession with a view to the removal of all existing evils which tend to diminish the birth-rate in these islands.

The *Johns Hopkins Hospital Bulletin* contains some interesting facts collected by Dr. Joseph Walsh on tuberculosis work in Europe. Dr. Walsh's researches convince him that sufficient rest, sufficient fresh air, and sufficient good nourishment are the only valuable elements in the treatment of tuberculosis as at present known to science. Dr. Walsh contrasts the uses of the system of treatment in sanatoria, as shown by the methods of treatment in various countries. The Germans hold the purpose of a sanatorium to be (1) the cure of the patient; (2) the education of the patient in the way of prevention in order to avoid further contagion; (3) the prevention of further contagion on the part of the patient for the time he is in the sanatorium. In England the purpose of the sanatorium is stated to be merely educational, but we cannot accept this view as correct. France, with her natural antagonism to Germany, opposed sanatoria until ten years ago. The French have directed all their efforts to the prevention of tuberculosis in the child, and to the treatment of the child. There are more sanatoria for children in France than in all other countries combined. The United States consider the purpose of the sanatorium twofold: (1) the cure of the patient, (2) the education of the patient and his friends. Dr. Walsh visited most of the private sanatoria in Germany, and his report would seem to show that although many of these establishments are magnificently equipped, the treatment in them is nothing like so good for the patients as it is in the public sanatoria.

